# Tracheostomy reversal years after patient lost to follow-up

**DOI:** 10.1186/s40463-018-0291-x

**Published:** 2018-07-13

**Authors:** Jill Querney, Jean-Luc Ethier

**Affiliations:** 1 Timmins and District Hospital, Northern Ontario School of Medicine, 935 Ramsey Lake Road, Sudbury, ON P3E 2C6 Canada; 2 University of Alberta, 8440 112 St NW, Edmonton, AB T6G 2B7 Canada

**Keywords:** Tracheostomy, Decannulation, Rural health

## Abstract

**Background:**

Pediatric tracheostomies occur for various reasons, including prologned intubation, and require a multidisciplinary approach with routine follow-up.

**Case presentation:**

This report reviews the history and clinical outcome of a 29 year old female patient who was lost to follow-up for nearly two decades after a pediatric tracheostomy. When she presented to the Otolaryngology service as an adult the original indication for tracheostomy had resolved and decannulation was initiated, but a profound psychological dependence had developed.

**Conclusion:**

This case outlines the importance of regular follow-up for tracheostomy patients, as well as health care barriers faced in remote rural communities.

## Background

The 29-year-old female patient under discussion lived with a tracheostomy for over two decades. The patient has spina bifida and experienced a complication after back surgery at age 6 that resulted in a blocked ventriculoperitoneal (VP) shunt and subsequent hydrocephalus. She had compromised vocal cord function, presumably secondary to a Chiari I malformation, and she received a tracheostomy after prolonged intubation. Unfortunately, the patient did not have follow-up care for the tracheostomy for eighteen years.

Symptom resolution post tracheostomy secondary to Chiari I malformations has been documented in the literature [1, 2]. The case being reviewed underscores the importance of routine follow-up care for pediatric patients with tracheostomies. In the absence of follow-up, significant morbidity and psychological dependence can develop, both of which are potentially avoidable.

Tracheostomy care consensus statements have been published with an over-arching emphasis on a multidisciplinary approach, as well as noting the importance of patient and family education prior to tracheostomy procedures and subsequent discharge from the hospital [3–5]. Additionally, the American Thoracic Society has made clear the importance of involving a speech language pathologist, as well as appropriate decannulation procedures to be followed for chronic tracheostomies [4]. Pediatric decannulation protocols have suggested operative endoscopy and removal of suprastromal granulation tissue, without advocating for routine capping or downsizing of tracheostomy tubes [6].

### Case Presentation

The patient is from a rural community ten hours from the nearest pediatric tertiary care hospital. She was born with spina bifida and several medical comorbidities requiring follow up at a pediatric tertiary care hospital, namely VP shunts for hydrocephalus and a repair of an Arnold Chiari I malformation during early childhood.

The patient had back surgery at the age of 6, and during the post-operative period had several respiratory arrests and was transferred back to the tertiary center. Her VP shunt had been disconnected, leading to worsening hydrocephalus and secondary reactivation of Chiari type symptomatology with bilateral vocal cord paralysis. She subsequently received a tracheostomy for prolonged intubation, as well as a gastrostomy tube due to dysphagia and aspiration.

The two and a half month hospital stay was very difficult for the family, both due to the stress placed on the family with regards to the patient’s health, and financially as they travelled ten hours by car and could not return home. After two months in hospital the patient’s mother signed a document stating she was taking full responsibility for her daughter’s health and they were discharged home against medical advice. No follow-up appointments were organized for the tracheostomy, although they still had annual follow-up for other medical conditions at this institution.

Over the years, the patient’s breathing and eating improved significantly. At the age of 22, the patient was able to eat small amounts of food without evidence of aspiration through the tracheostomy. Although the patient was deemed to be a fairly high risk of aspiration, she only had pneumonia diagnosed once during her tracheostomy period. While the patient had a weak voice, her speech continued to improve.

The reversal of the tracheostomy was a very long transition. The first referral to the senior author was made in 2010, and the physical exam of the patient was significant for mobile vocal cords with an anterior glottic gap. The tracheostomy was occluded in the office and the patient was able to breath with improved velopharyngeal closure and cord mobility. A plan was therefore developed to wean the patient from her tracheostomy. A barium swallow and videofluoroscopy revealed that the patient had difficulties with the oral and pharyngeal phases of swallowing, and that she was at moderate risk for complications secondary to dysphagia. The possibility that the tracheostomy might be worsening the patient’s swallowing function, as well as the risk of oral intake, were clearly reviewed with the patient and her family. A second opinion from a laryngologist was obtained and supported the opinion that decannulation was indicated.

The reversal process was delayed as a result of the family deferring follow-up care for a couple of years. When the patient eventually returned, a sleep study conducted reported no respiratory distress with the tracheostomy corked and a normal apnea-hypopnea index. From that point forward, the patient began to sleep with her tracheostomy corked, and the family was encouraged to stop suctioning completely. Just over one year later, decannulation and closure of the tracheostoma site was completed under local anesthetic and tolerated extremely well. Following the procedure, the facial expression on the patient’s face alone was worth every second of work and counseling done by the senior author!

## Discussion

This unnecessarily prolonged use of a tracheostomy underlies the importance of follow-up, and the unfortunate outcomes that can result when patients are lost to follow-up care. Furthermore, health care in rural and remote areas can be complicated by the travel requirements for specialist appointments.

The care and medical management of chronic pediatric tracheostomies has been previously outlined, with importance placed on a multidisciplinary approach, and recommended protocols for follow up care place an emphasis on family education [3, 4, 6–8]. We endorse these recommendations and in addition advocate for the inclusion of a discussion regarding the importance of follow-up appointments, appropriate inter-appointment intervals, and giving families an idea of what should be expected upon discharge.

Similar to much of the practice of medicine, cases such as this one aren’t straightforward; however, several social issues need to be addressed. To begin, the patient’s family was overwhelmed and they perceived a lack of support. Hospital programs to address these issues for families would be optimal. Many improvements have been implemented to support patients and their families since our patient’s experience; however, we still need to strive to help families despite the financial constraints in the health care system.

A burden of responsibility also lies with the patient’s primary caregivers with respect to taking her health in their own hands, and arranging for appropriate care and follow-up. While the form signed to discharge the patient against medical advice absolved the hospital of liability, it didn’t negate the need for follow-up care. It behooves us as physicians to take into consideration the potential long-term sequelae of patients acting against our recommendations and we need to ensure patients or their caregivers fully understand the situation and potential implications.

The reversal of the tracheostomy was a very prolonged process. The patient and her family had developed a severe psychological dependence toward the tracheostomy, and she had to make progress with respect to her dysphagia. Had time-appropriate follow-up occurred, the psychological aspect of this case might have been avoided or at least mitigated. Overall, the patient fell through the cracks in the health care system at multiple levels; and awareness surrounding the importance of follow-up care is essential to prevent history from repeating itself.

## Conclusion

Ensuring that follow-up appointments are arranged is essential for appropriate patient care, as is attending those appointments. Patients living in rural and remote areas have less access to specialists, and may require more involvement and oversight to arrange all necessary follow-up appointments. The above case outlines the potential downfalls of our system when a patient slips through the cracks as well as the importance to try seal these cracks.
